# Implications of navigation system use for glenoid component placement in reverse shoulder arthroplasty

**DOI:** 10.1038/s41598-022-25833-8

**Published:** 2022-12-07

**Authors:** Hiroaki Kida, Atsushi Urita, Daisuke Momma, Yuki Matsui, Takeshi Endo, Daisuke Kawamura, Hiroshi Taneichi, Norimasa Iwasaki

**Affiliations:** 1 Department of Orthopaedic Surgery, Faculty of Medicine and Graduate School of Medicine, Hokkaido University, Sapporo, Japan; 2 Department of Orthopaedic Surgery, Dokkyo Medical University, Kitakobayashi 880, Mibu, Tochigi 321-0293 Japan; 3 Center of Sports Medicine, Hokkaido University Hospital, Sapporo, Japan

**Keywords:** Medical research, Engineering

## Abstract

Recently, three-dimensional (3D) planning, patient-specific instruments, and navigation system have been developed to improve the accuracy of baseplate placement in reverse shoulder arthroplasty (RSA). The purpose of this study was to evaluate baseplate placement using the navigation system. Sixty-four shoulders in 63 patients who underwent RSA for rotator cuff tear arthropathy or irreparable rotator cuff tears were enrolled. Conventional RSA was performed in 31 shoulders and navigated RSA using pre-operative planning software was performed in 33 shoulders. The use of augmented baseplates, the version and inclination of the baseplate, and screw length were compared between conventional RSA and navigated RSA. Augmented baseplates were used more frequently in navigated RSA than in conventional RSA (20 vs 9 shoulders, p = 0.014). Baseplate alignment was 1.0° (SD 5.1) of retroversion and 2.4° (SD 6.8) of superior inclination in conventional RSA and 0.2° (SD 1.9) of anteversion and 0.3° (SD 1.7) of superior inclination in navigated RSA. Compared with conventional RSA, precision of baseplate version and inclination were higher in navigated RSA (both p < 0.001). Superior, inferior, and posteroinferior screws were significantly longer in navigated RSA than in conventional RSA (p = 0.021, 0.001 and < 0.001, respectively). Precision of superior and inferior screw lengths was significantly higher in navigated RSA than in conventional RSA (both p = 0.001). Our results suggest that adoption of pre-operative planning software increased augmented baseplate use to minimize the glenoid reaming. The navigation system allows placement of the baseplate accurately, according to the pre-operative plan. Furthermore, the navigation system enables monitoring of screw length and direction in real time.

## Introduction

Baseplate stability and alignment are associated with clinical outcomes after reverse shoulder arthroplasty (RSA)^1,2^. Poor positioning of the baseplate leads to various complications in RSA^3–9^. Failure of the glenoid component due to instability and loosening is the most common complication requiring revision^2^. Excessive version or inclination of the glenoid component can lead to micromotion and stress concentration that results in bone resorption and loosening^4–6^. In addition, excessive reaming of the glenoid sacrifices supportive subchondral bone to correct glenoid alignment, which causes subsidence of the component^10^.

Computer-assisted surgical technology, including three-dimensional (3D) planning software, patient-specific instrument (PSI) guides and a navigation system have been developed to improve the accuracy of glenoid component placement in shoulder arthroplasty^11–15^. Pre-operative planning software enables selection and positioning of a desired size of glenoid component to ensure baseplate backside support. PSI guides and navigation systems have become widely used for placement of the glenoid component as planned pre-operatively^11,13^. Furthermore, use of a navigation system enables real-time visualization of screw direction as well as baseplate alignment.

The purpose of this study was to clarify the accuracy and precision to execute the surgical plan of the baseplate placement in RSA with the navigation system and pre-operative planning software compared to RSA with conventional instrumentation. We hypothesized that (1) pre-operative planning software would enable the selection of proper type of baseplates and thus increase the number of augmented baseplates used, and (2) the navigation system would enable placement of the baseplate component as planned, and the insertion of longer screws for baseplate fixation.

## Materials and methods

### Study design

This retrospective study included 71 patients who underwent RSA for treatment of cuff tear arthropathy and irreparable rotator cuff tear between November 2017 and October 2020. Of these, RSA with the conventional method was planned in 32 shoulders (conventional RSA) and that with the navigation method was planned in 39 shoulders (navigated RSA). Four shoulders in the navigated RSA group were changed to the conventional method (navigation error, n = 2; coracoid tracker loosening, n = 2). Both were not able to register points on the coracoid and glenoid correctly. It is avoidable because these were considered to be technical errors in initial cases. Five shoulders in the conventional RSA group and 2 shoulders in the navigated RSA group were lost to follow-up and excluded from the study. A final total of 31 shoulders in the conventional RSA group and 33 shoulders in the navigated RSA group were analysed.

Patient characteristics including age at surgery, sex, operative side, range of motion, operative time, intra-operative blood loss, the use of glenoid component option, and screw length for baseplate fixation were recorded. Pre-operative clinical outcomes were assessed using the 48-point Oxford shoulder score and the 100-point Constant score. Pre-operative glenoid morphology (erosion pattern) was evaluated using computed tomography (CT) according to Walch classification in the axial plane and Favard classification in the coronal plane^16,17^.

### Pre-operative planning

**Figure 1:**
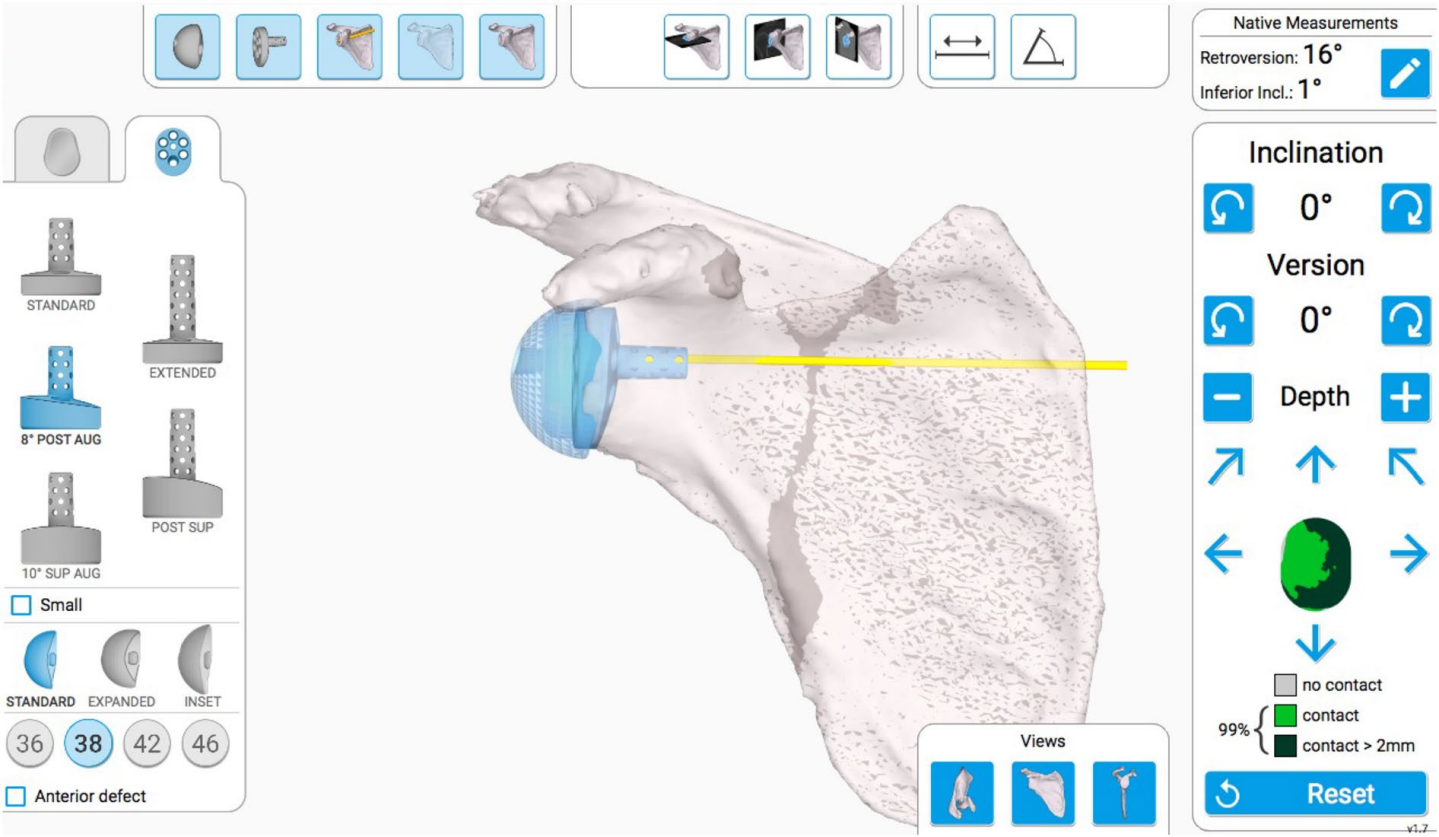
Pre-operative planning software of the Equinoxe Planning App. Virtual glenoid baseplate placement is used to determine the ideal component type and its position. *Post* posterior, *Aug* augmented, *Sup* superior.

All patients underwent RSA with the Exactech Equinoxe system (Exactech, Gainesville, FL, USA), which has the design of a medialized glenoid and lateralized humerus. Glenoid implant options included standard, 8° posterior augment, and 10° superior augment. In the case of glenoid erosion, an augmented glenoid baseplate component was used to correct version and inclination. In conventional RSA, positioning of the central peg of the baseplate was determined in 2D planning based on radiographs and CT images without pre-operative planning software. In navigated RSA, the Equinoxe Planning App pre-operative planning software (Exactech Blue Ortho, Grenoble, France) was used. CT images of the shoulder joint were acquired in the axial plane in 0.625-mm thick contiguous slices. After segmentation of the scapula, Friedman’s axis was determined from the centre of the glenoid to the medial border of the scapula^18^. In the planning software, a virtual glenoid baseplate was placed onto the 3D model of the scapula (Fig. 1). The position of the baseplate was decided along Friedman’s axis at 0° of version and 0° of inclination. When correction of glenoid version or inclination required excessive reaming, an augmented baseplate was used to minimize the amount of bone removal. The surgical plan was then saved and uploaded to the intra-operative navigation system.

### Surgical procedure

**Figure 2:**
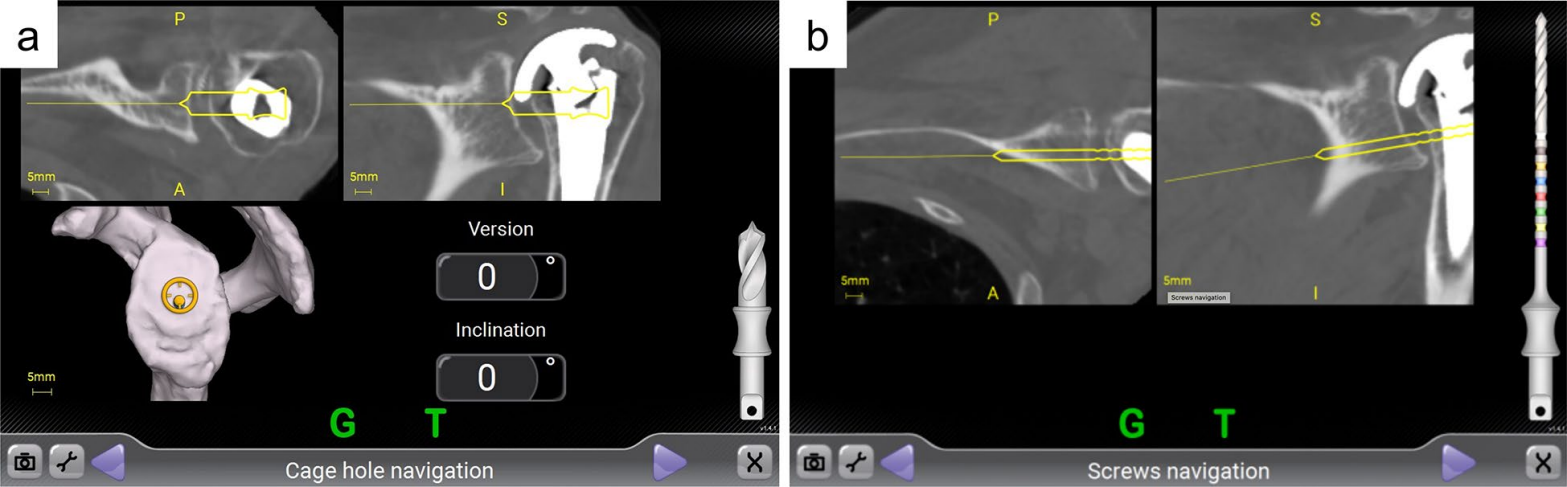
The Exactech GPS navigation screen. (**a**) The central peg hole shows 0° of anteversion error and 0° of superior inclination error in comparison with the pre-operative plan. (**b**) The drill bit direction is confirmed for inferior screw insertion with real-time monitoring. *P* posterior, *A* anterior, *S* superior, *I* inferior.

The standard deltopectoral approach was performed in both groups. After humeral head osteotomy, the glenoid surface was exposed. In conventional RSA, the glenoid centre was determined at the intersection of the vertical and perpendicular midlines of the glenoid. A 2.0 mm Kirschner wire was inserted in neutral version and inclination, with visual inspection. Reaming, baseplate central peg hole creation, and screw insertion for baseplate placement were then performed manually. In navigated RSA, the tracker was placed on the coracoid. Several points on the coracoid and glenoid were registered using the pointer of the tracker and then linked to the planning model. Glenoid reaming and drilling of the central peg hole were performed according to the pre-operative plan (Fig. 2a). The baseplate was then fixed onto the glenoid. The baseplate has six screw holes available with variable-angle screw fixation, of which four (superior, inferior, anteroinferior, and posteroinferior) were used in all cases. In navigated RSA, the navigation screen displays the trajectory of the drill bit on coronal and sagittal images in real time (Fig. 2b). Locking caps were placed on the screw heads prior to glenosphere placement. After humeral reaming and broaching were performed, the humeral components were placed.

### Measurements

**Figure 3:**
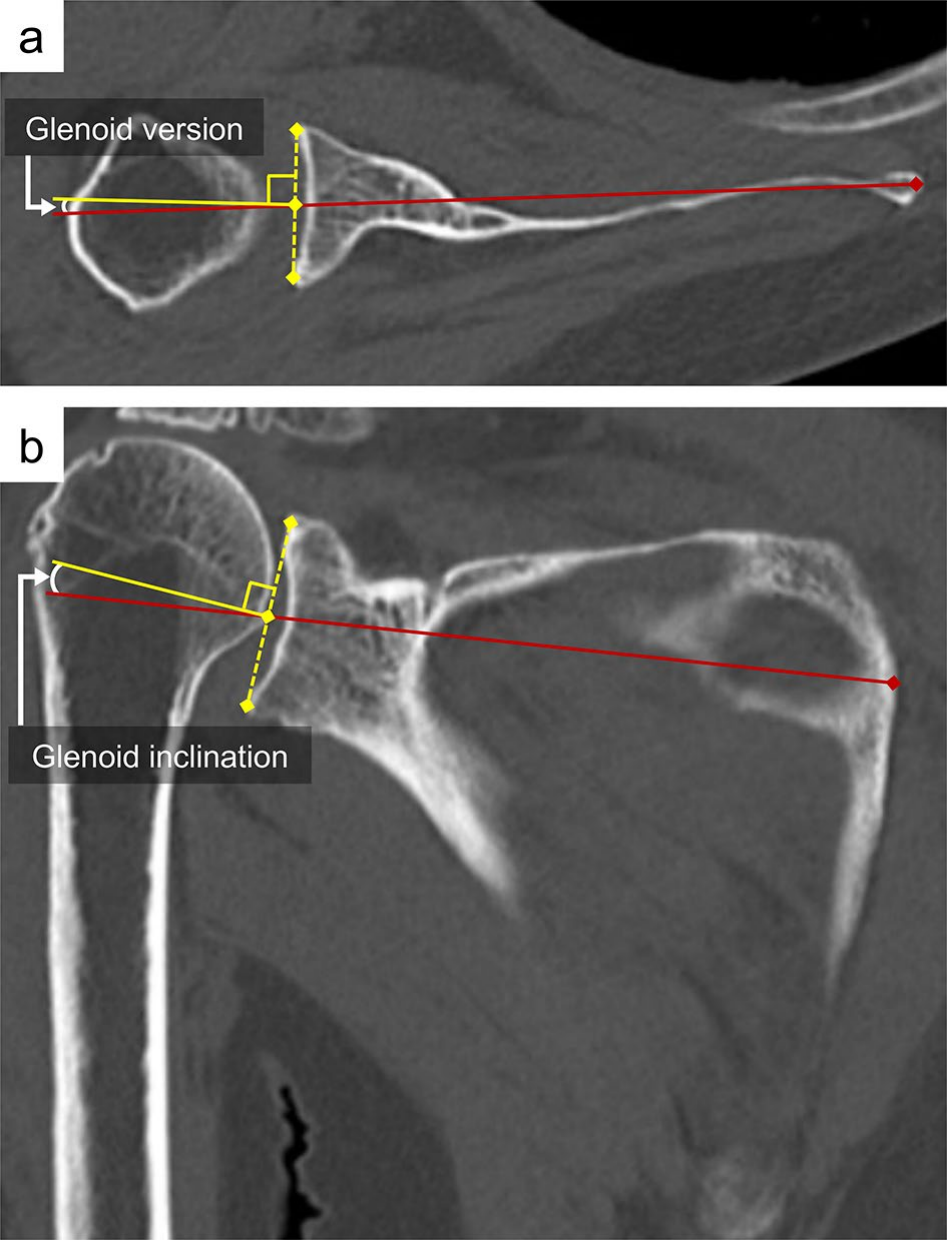
Pre-operative measurement of glenoid version and inclination. (**a**) Glenoid version was defined as the angle between Friedman’s axis and a line perpendicular to the line between the anterior and posterior rims of the glenoid. (**b**) Glenoid inclination was defined as the angle between Friedman’s axis and a line perpendicular to the line between the superior and inferior rims of the glenoid.

**Figure 4:**
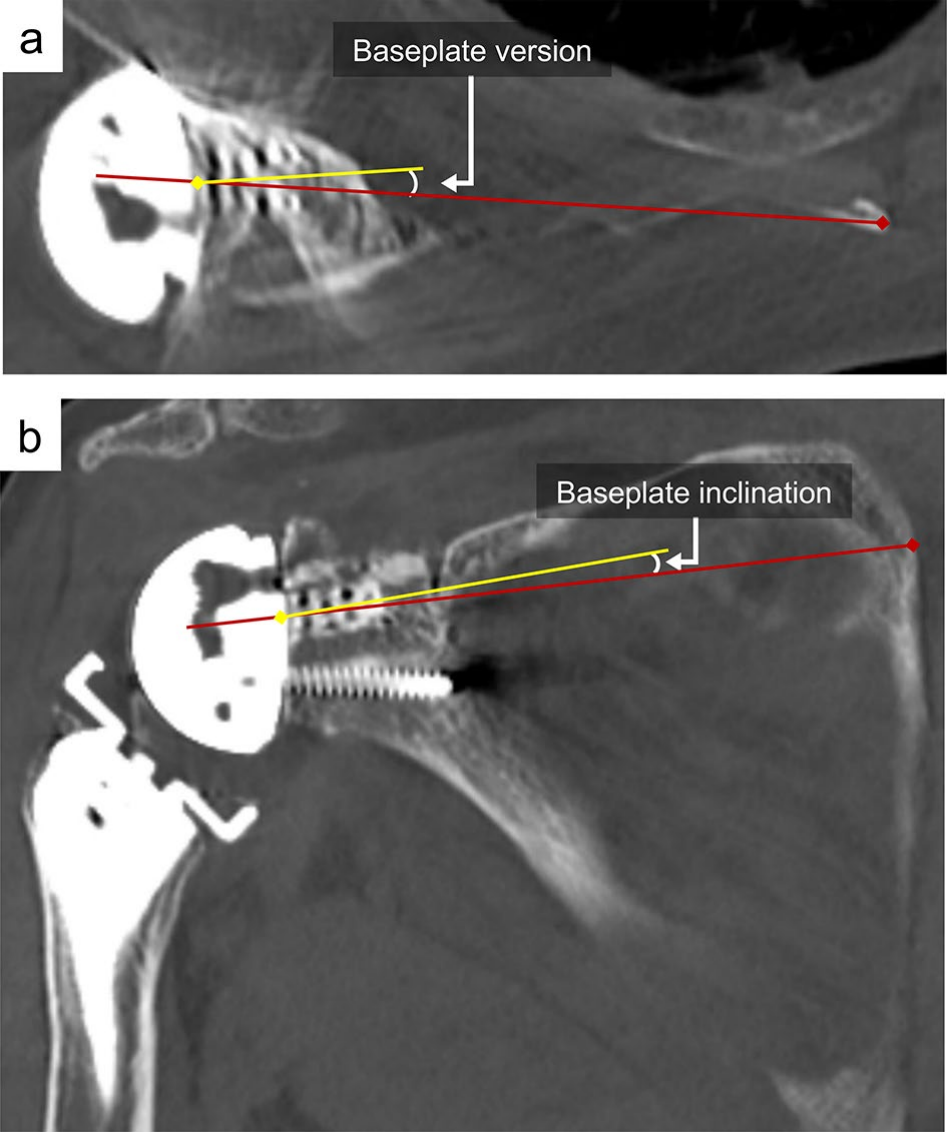
Measurement of glenoid component alignment including version and inclination. (**a**) Baseplate version was defined as the angle between Friedman’s axis and the axis of the central cage. (**b**) Baseplate inclination was defined as the angle between Friedman’s axis and the axis of the central cage.

All patients underwent CT scans pre-operatively and at one week post-operatively. All parameters were measured three times by 1 author (H.K.), who was blinded to the patient information of study groups and the mean value was used in the analysis. Glenoid morphology was evaluated based on angles of version in the axial plane and inclination in the coronal plane (Fig. 3). The alignment of the baseplate on the glenoid was evaluated by measurement of version in the axial plane and inclination in the coronal plane (Fig. 4). Positive and negative values of version were considered to indicate anteversion and retroversion, respectively. On the other hand, positive and negative values of inclination were considered to indicate superior and inferior inclination, respectively.

### Statistical analysis

The data were analysed using JMP software (v12.0, SAS Institute, Cary, NC) and G*Power Software (v3.1.9.6, Heinrich-Heine-Universität Düsseldorf, Düsseldorf, Germany). Intraclass correlation coefficient was used to evaluate intraobserver reliability for measurements of pre-operative glenoid alignment and baseplate alignment. An intraclass correlation coefficient of 0 to 0.4 indicates low reliability, > 0.4 to 0.75 indicates fair reliability, and > 0.75 indicates excellent reliability^19^. All variables were tested for normal distribution. Patient demographic data including age, range of motion, clinical scores, operative time, intra-operative blood loss, and pre-operative glenoid morphology were compared between the groups using unpaired t-test. Sex, operative side, augmented baseplate use, and the distribution of glenoid type (including Walch classification and Favard classification) were compared between the groups by χ^2^ test. To evaluate the efficacy of the navigation system, unpaired t-test was used to compare baseplate alignment and screw length between the groups. Furthermore, precision of baseplate alignment and screw length was compared using an F test. Fisher’s exact test was used to compare the percentage of baseplates placed within 5° or at 8° or more of the pre-operative plan between the groups. A p-value < 0.05 was considered to indicate statistical significance. To assess the relevance of number of cases, posteriori power analysis was conducted using the observed variances, sample size and an alpha of 0.05 to calculate posteriori power (1 − β) for F tests.

### Ethical approval

Ethics approval for this study was provided by the Institutional Review Board of Hokkaido University School of Medicine (##017-0522), and written informed consent was obtained from all patients. The methods were carried out in accordance with the principles of the Declaration of Helsinki.

## Results

### Patient demographics

There was no significant difference in patient demographics between the groups (Table 1). There were no intra-operative complications in both groups. Table 2 lists the obtained measurements of glenoid morphology. There was no significant difference between the groups in terms of version or inclination (p = 0.571 and p = 0.612, respectively), or in the distribution of glenoid type such as Walch classification or Favard classification (p = 0.359 and p = 0.286, respectively).

**Table1:** Patient demographics.

	Conventional RSA	Navigated RSA	p-value
N	31	33	
Mean age, years (SD)	74.5 (6.1)	75.2 (6.4)	0.662
Sex, n, male/female	13/18	14/19	0.317
Operative side, n, right/left	20/11	23/10	0.791
**Mean range of motion**			
Flexion, ° (SD)	56.6 (34.8)	57.7 (28.5)	0.891
Abduction, ° (SD)	57.1 (35.6)	54.1 (28.2)	0.725
External rotation, ° (SD)	15.5 (17.8)	10.8 (15.8)	0.299
Internal rotation*, points (SD)	4.5 (1.7)	5.3 (2.2)	0.141
Mean Oxford shoulder score, points (SD)	13.7 (3.9)	15.4 (4.6)	0.221
Mean constant score, points (SD)	22.1 (11.1)	23.7 (13.9)	0.687
Mean operative time, minutes (SD)	125.0 (37.8)	137.1 (31.7)	0.169
Mean intra-operative blood loss, ml (SD)	125.9 (146.5)	178.9 (141.6)	0.315

*n* number, *SD* standard deviation. *Internal rotation: In accordance with the Constant score, 0, lateral thigh; 2, buttock; 4, lumbosacral junction; 6, L3 (waist); 8, T12; 10, T7 (interscapular). p < 0.05 is considered statistically significant.

**Table 2:** Glenoid morphology.

	Conventional RSA	Navigated RSA	p-value
Mean version, ° (SD)	3.8 (7.7)	4.8 (5.5)	0.571
Reliability (ICC)	0.98	0.98	
Mean inclination, ° (SD)	4.4 (4.8)	3.8 (3.5)	0.612
Reliability (ICC)	0.98	0.98	
**Walch classification, n**			0.359
A1/A2	18/2	17/1	
B1/B2/B3	8/1/1	11/4/0	
C	0	0	
D	1	0	
**Favard classification, n**			0.286
E0	18	22	
E1	4	2	
E2	6	3	
E3	2	6	
E4	1	0	

*SD* standard deviation, *ICC* intraclass correlation coefficient, *n* number. p < 0.05 is considered statistically significant.

### Selection of augmented baseplate

Augmented baseplates were used significantly more frequently in the navigated RSA group (n = 20, 60.6%) than in the conventional RSA group (n = 9, 29.0%) (p = 0.014). In conventional RSA, a superior augmented component was used in 3 shoulders and a posterior augmented component was used in 6 shoulders. In navigated RSA, a superior component was used in 5 shoulders and a posterior component was used in 15 shoulders.

### Baseplate alignment

**Figure 5:**
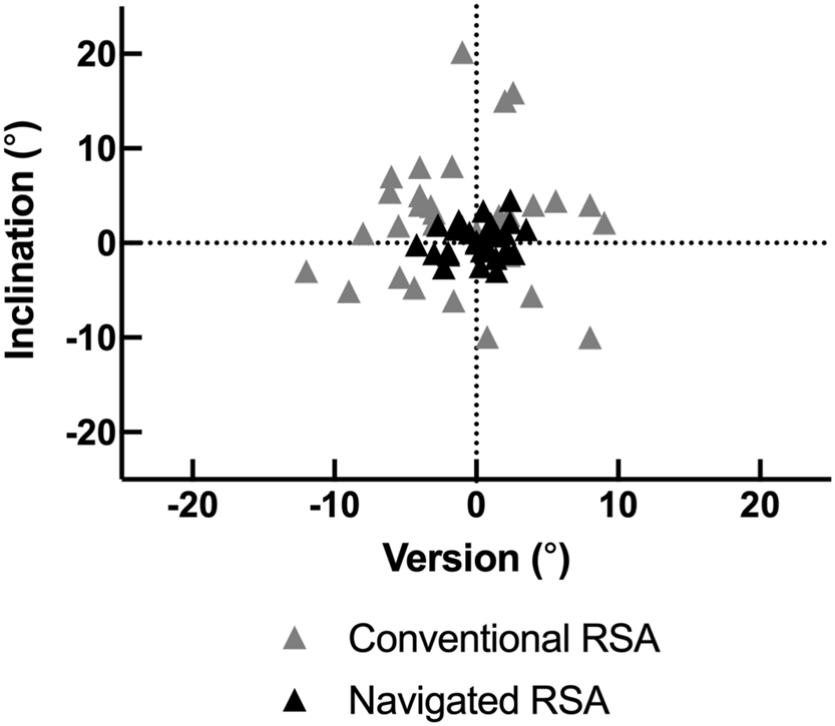
Distribution of the glenoid baseplate version and inclination in conventional RSA (grey triangles) and navigated RSA (black triangles).

Although there was no significant difference in version and inclination between the groups (p = 0.238 and p = 0.093, respectively), precision was significantly higher in both version and inclination in navigated RSA compared with conventional RSA (both p < 0.001) (Table 3, Fig. 5). There were significantly more cases of baseplate alignment within 5° of the pre-operative planning in navigated RSA than in conventional RSA (100% vs 64.5%, p = 0.001 in version and 100% vs 61.3%, p = 0.001 in inclination). There were significantly fewer cases of baseplate alignment of 8° or more from the pre-operative planning in navigated RSA than in conventional RSA (0% vs 19.4%, p = 0.010 in version and 0% vs 22.6%, p = 0.004 in inclination).

**Table 3:** Baseplate alignment and Mean screw length for baseplate fixation.

	Conventional RSA	Navigated RSA	p-value unpaired t-test	p-value F test	1 − β
**Baseplate alignment**					
Mean version, ° (SD)	− 1.0 (5.1)	0.2 (1.9)	0.238	**< 0.001**	0.99
Reliability (ICC)	0.98	0.98			
Mean inclination, ° (SD)	2.4 (6.8)	0.3 (1.7)	0.093	**< 0.001**	0..99
Reliability (ICC)	0.97	0.98			
**Mean screw length**					
Superior screw, mm (SD)	28.3 (5.8)	31.0 (3.2)	**0.021**	**0.001**	0.90
Inferior screw, mm (SD)	29.4 (6.2)	33.5 (3.4)	**0.001**	**0.001**	0.90
Anteroinferior screw, mm (SD)	23.5 (3.8)	24.7 (4.2)	0.271	0.601	0.08
Posteroinferior screw, mm (SD)	21.2 (3.5)	27.7 (4.8)	**< 0.001**	0.083	0.41

*SD* standard deviation, *ICC* intraclass correlation coefficient. Significant values are given in bold. p < 0.05 is considered statistically significant.

### Screw purchase for baseplate fixation

Four screws (superior, inferior, anteroinferior, and posteroinferior) were used in all cases. Superior, inferior, and posteroinferior screws in the navigated RSA were significantly longer than those in the conventional RSA (p = 0.021, 0.001, and < 0.001, respectively) (Table 3). While there was no significant difference in terms of precision of anteroinferior and posteroinferior screws (p = 0.601and p = 0.083, respectively), precision of superior and inferior screw length was significantly higher in navigated RSA than in conventional RSA (both p = 0.001) (Table 3).

## Discussion

The purpose of this study was to clarify the implications of pre-operative planning software and use of an intra-operative navigation system for placing the baseplate component in RSA. Our results showed that the number of augmented baseplates used was greater with pre-operative planning software. Precision of baseplate alignment was higher in navigated RSA than in conventional RSA. There were more cases of baseplate alignment within 5° of the pre-operative planning and fewer cases of baseplate alignment of 8° or more in navigated RSA. Furthermore, screws were longer and more precise in navigated RSA. These results confirm the usefulness of the pre-operative planning software and navigation system used in RSA.

Poor positioning of the baseplate can increase the risk of various complications in RSA. Excessive superior inclination leads to aseptic loosening, scapular impingement, instability, inferior scapular notching, and medial polyethylene wear^6–8^, whereas inferior inclination of more than 10° leads to increased micro-motion and shear force on the baseplat^5^. In contrast, more than 10° of retroversion risks stress concentration on the glenoid and posterior glenoid bone resorption^4^. Moreover, retroversion of more than 15° increases osteolysis around the glenoid centre peg^9^. To prevent such complications, computer-assisted surgical technology, including 3D planning software, PSI guides, and intra-operative navigation systems have been developed to achieve proper baseplate alignment.

Pre-operative planning software is increasingly being used for the accurate evaluation of glenoid morphology and to enable selection of the appropriate glenoid component^15^. Although excessive reaming of the glenoid surface is sometimes required to correct a glenoid deformity, this causes a decrease in glenoid bone stock and medializes the centre of rotation, which results in poor fixation of the component, loss of range of motion and muscle strength, and instability^20^. The augmented baseplate minimizes scapular bone removal and maximizes correction of glenoid deformity^21^. In the present study, use of pre-operative planning software was associated with increased use of augmented baseplates. This finding suggests that pre-operative templating of baseplate in planning software enabled the selection of more proper type of baseplates and allowed the augmented baseplate to be placed without excessive reaming or overly medializing the centre of rotation. However, augmented baseplates may cause excessive lateralization of the glenoid component, leading to decreased range of motion^22^.

The PSI guide is custom-made for placing the baseplate component, based on pre-operative planning with 3D CT images. This instrument reduces deviation from the intended position in version and inclination^12,14^. However, Simon et al. reported that the in vivo accuracy of PSI-guided glenoid positioning is not as successful as suggested in the literature^23^. The meta-analysis of Cabarcas et al. also reported no significant differences in accuracy between PSI and standard instrumentation^24^. Furthermore, PSIs take time and cost to make after planning and are not used in all patients^25^.

The intra-operative navigation system can immediately utilize data obtained in planning placement of the baseplate. Several studies have investigated the effectiveness of glenoid component placement in shoulder arthroplasty^11,26,27^. Kircher et al. reported baseplate retroversion of 3.7° (SD 6.3) with navigation, whereas baseplate retroversion was 10.9° (SD 6.8) without navigation^26^. Nashikkar et al. reported that the incidence of neutral inclination or version was high when navigation was used in shoulder arthroplasty including anatomic total shoulder arthroplasty and RSA, and the baseplate was implanted within 5° of the surgical plan in > 70% of cases^11^. Similarly, our results showed higher precision of baseplate alignment in navigated RSA than in conventional RSA. This result suggests that the navigation system can reliably place the baseplate component as planned.

As an additional value of the navigation system for RSA, navigation enables monitoring of the direction of screw insertion in real time. Initial fixation of the baseplate is essential for long-term stability^28–30^. Longer baseplate screws have been demonstrated to improve glenoid baseplate fixation^31,32^. The inferior screw is the most important for fixation because the tensile load is applied to the inferior aspect of the glenoid prosthesis^33^. The present navigation system enabled the use of longer screws, to obtain stronger primary fixation strength.

Longer screws directed between the 2-o’clock and 8-o’clock positions on the glenoid cause a risk of suprascapular nerve injury^31,34^. In addition, improper superior screw direction causes acromion fractures after RSA^35^. The navigation system allows screw insertion in a safe area by monitoring the direction of screw insertion in real time. Gavaskar et al. reported that the use of navigation may reduce the risk of acromion fractures by preventing perforation by the upper screw^36^. Our findings showed that the navigation system improves the precision of screw length and enables use of constant screw length while monitoring the safe zone of screw direction.

There were several limitations in the current study. First, the study design was nonrandomized. There is a possibility of measurement bias in differences between the conventional and navigation methods. However, blinded analysis was performed in this study, in which the digital images were measured without knowledge of the patient information of study groups. Therefore, measurement bias in the results was suppressed to some extent. A prospective randomized study is needed in the future to overcome this limitation. Second, there were few cases of severe glenoid erosion in this study. The classifications of Walch A1 and Favard E0 do not require aggressive correction of glenoid version or inclination. Therefore, the effectiveness of the navigation system may be further clarified when evaluating cases of severe glenoid bone erosion. Third, while not significantly different, navigated RSA was observed to take 12.1 min longer than conventional RSA. Longer operative time can increase risks of peri-operative complications. However, operative time can decrease as the number of cases increases because it takes getting used to the navigation system. Finally, the correlation between the use of navigation and clinical outcomes for RSA is currently unclear. As the navigation system is more costly than conventional planning, it must be highly cost-effective. In the future, additional studies are needed to clarify the effect of the navigation system on clinical outcomes and implant survivorship. Despite these limitations, this study demonstrated improvements in execution of the surgical plan with navigated RSA as compared to conventional RSA.

## Conclusion

This study investigated the implications of pre-operative planning software and a navigation system for glenoid component placement in RSA. The pre-operative planning software increased the number of augmented baseplates used to minimize the glenoid reaming. The navigation system enables the surgeon to more accurately and precisely reproduce baseplate placement as planned pre-operatively as compared to conventional instrumentation. Furthermore, the navigation system enables real time monitoring of the direction and the use of longer screws. Further studies are necessary to clarify implant survivorship and clinical implications of the navigation system in RSA.

## Data Availability

The datasets used and/or analysed during the current study are available from the corresponding author on reasonable request.
